# Safety and Effectiveness of Cinnomer^®^ on Disease Characteristics, Depression, and Quality of Life of Patients with Multiple Sclerosis: A Phase IV, Post-marketing, Prospective, Multicenter Study

**DOI:** 10.34172/aim.2023.95

**Published:** 2023-11-01

**Authors:** Abdorreza Naser Moghadasi, Fereshteh Ashtari, Seyed Mohammad Baghbanian, Vahid Shaygannejad, Nassim Anjidani, Fereshteh Ghadiri, Behnaz Sedighi, Morteza Saeidi, Hamed Amirifard, Hormoz Ayromlou, Nahid Beladi Moghadam, Mohammad Bagher Ranjbar, Masoume Nazeri, Zahra Niknam, Fardin Faraji, Afsaneh Afsorde, Mohammad Ali Sahraian

**Affiliations:** ^1^Multiple Sclerosis Research Center, Neuroscience Institute, Tehran University of Medical Sciences, Tehran, Iran; ^2^Isfahan University of Medical sciences, Kashani Comprehensive MS Center, Isfahan, Iran; ^3^Multiple Sclerosis Fellowship, Neurology Department, Boualicina Hospital, Mazandaran University of Medical Sciences, Sari, Iran; ^4^Medical Department, Orchid Pharmed Company, Tehran, Iran; ^5^Neurology Research Center, Kerman University of Medical Science, Kerman, Iran; ^6^Department of Neurology, Ghaem Hospital, Mashhad University of Medical Sciences, Mashhad, Iran; ^7^Department of Neurology, Imam Khomeini Hospital Complex, Tehran University of Medical Sciences, Tehran, Iran; ^8^Neurology Department, Imam Reza Hospital, Tabriz University of Medical Sciences, Tabriz, Iran; ^9^Department of Neurology, Shahid Beheshti University of Medical Sciences, Tehran, Iran; ^10^Pastour Building, Shiraz, Iran; ^11^Clinical Neurology Research Center, Department of Neurology, Shiraz University of Medical Sciences, Shiraz, Iran; ^12^Kosar Hospital, Shiraz, Iran; ^13^Department of Neurology, School of Medicine, Arak University of Medical Sciences, Arak, Iran; ^14^Sina Building, Shiraz, Iran

**Keywords:** Cinnomer^®^, Clinical trial phase IV, Glatiramer acetate, Multiple sclerosis, Quality of life

## Abstract

**Background:**

Every patient diagnosed with definite multiple sclerosis (MS) should begin disease modifying therapies. Cinnomer^®^ contains 40 mg glatiramer acetate (GA) and is available in prefilled syringes and autoinjector devices.

**Methods:**

A phase IV multicenter study was conducted to explore the safety and effectiveness of Cinnomer^®^ in the treatment of MS. Study-related data were collected for 14 months.

**Results:**

Totally, 368 Iranian relapsing-remitting MS patients in nine cities were enrolled. The patients were either treatment naïve (n=191) or switchers (n=177). Cinnomer^®^ treatment was associated with a significant reduction in annual relapse rate (ARR) (RR: 0.65, 95% CI: 0.43, 0.98). Final mean Expanded Disability Status Scale (EDSS) scores showed improvement from baseline (difference: -0.21, 95% confidence interval (CI): -0.34, -0.08). There was a significant decrease in gad-enhancing lesions during treatment (difference: -0.38, 95% CI: -0.64, -0.12). The mean score for the depression measure (21-item BDI-II questionnaire) significantly improved (difference: -2.39, 95% CI: -3.74, -1.03). There was a significant change in the "psychological well-being" dimension (*P*=0.02) (in line with BDI-II scores) and "rejection" MusiQoL dimensions (*P*=0.04). The adverse events documented throughout the study were not unexpected for GA and were principally not serious.

**Conclusion:**

Safety measures were in line with the known profiles of GA. The results suggest that Cinnomer^®^ is effective with respect to clinical outcomes and from the patient’s perspective and in reducing MRI-measured MS activity.

## Introduction

 Multiple sclerosis (MS) is a chronic autoimmune disease of the central nervous system (CNS) that targets the myelinated axons.^1^ Apart from treating acute MS attacks, consisting predominantly of high doses of corticosteroids and plasma exchange, the majority of those diagnosed with definite MS should begin one of the disease-modifying therapies (DMTs).^2^ Disease-modifying medications could decrease relapse rate, slow the accumulation of brain lesions and also delay disability progression.^3^

 Glatiramer acetate (GA) is an immune modulator, first found in 1960s, and approved by the FDA in 1996 as the first DMT for relapsing-remitting MS (RRMS). The alternate dose (40-mg/mL three times a week) was approved in 2014.^4^ Considering the rising costs of DMTs^4^ and their limited availability in countries like Iran, generic forms of such drugs may be of great help to patients and clinicians. Cinnomer^®^ (by CinnaGen) contains 40 mg GA and is available in prefilled syringes and autoinjector devices. Cinnomer^®^ is a clear, colorless to slightly yellow, sterile, nonpyrogenic solution for SC injection. Cinnomer^®^ has been used widely in the treatment of patients with MS in Iran since March 2015. Currently, 4257 cases are using this drug as their main DMT.

 Post-marketing studies are important to ensure drug safety and effectiveness and permit detection of less common but sometimes serious adverse events (AEs). Thus, in this phase IV multicenter study, we aimed to explore the safety and effectiveness of Cinnomer^®^ in the treatment of MS.

## Materials and Methods

###  Design

 To investigate the safety and effectiveness of Cinnomer^®^ in patients with MS in Iran, a single-arm phase IV prospective multicenter trial was designed. No control group was included.

###  Patients

 Patients with RRMS in 14 centers (from nine cities of Iran) were enrolled. The study population included treatment-naïve patients and switchers. Treatment-naïve patients were those without a history of receiving any treatment for MS. Switcher patients were those whose treatment was changed for any reason from one DMT to another. EDSS was between 0 and 5. Patients younger than 18 or older than 60 years were excluded. Also excluded were those with a history of hypersensitivity to GA, mannitol, or any component of the formulation. The patient was not entered or withdrew if the investigator believed that the subject was unsuitable for treatment with Cinnomer^®^. The occurrence of hypersensitivity to GA or any component of the formulation resulted in treatment withdrawal.

###  Variables

 The primary objective of this study was to assess the safety of Cinnomer^®^ in patients with RRMS. Therefore, in each visit, the AEs and their seriousness were recorded.

 The indicative parameters of MS activity, including relapse information, MRI findings, and Expanded Disability Status Scale (EDSS) scores were analyzed to explore the secondary objectives. Also, the patients’ quality of life (QoL) and depression were assessed. MusiQoL is a self-administered, multidimensional questionnaire, validated to assess the quality of life in MS patients.^5^ Beck Depression Inventory (BDI-II) is considered a fair test to evaluate depression in MS.^6^

###  Data Collection

 All the required data were collected through a comprehensive physical examination, complete blood cell count, MRI findings, and specific questionnaires (completed by patients) throughout the study by the specialists who visit the subjects. The study consisted of five visits, including a baseline screening visit performed before the trial and four additional follow-up visits. The first follow-up visit was in the second month after the study start date, and the remaining visits were conducted every four months.

 Participant’s data were recorded in paper-based case report forms termed PMS booklets. Two types of booklets were designed; booklet I contained the baseline visit information, and booklet II included the four additional follow-up visits. Physicians were asked to complete the safety and effectiveness information of Cinnomer^®^ use in patients in the designated booklets.

 An electronic data registry was developed based on the approved PMS booklet, and the investigators confirmed its conformity with the PMS booklet correspondingly. Patients’ data recorded in PMS booklets were monitored regularly in terms of verification and validity while the study was progressing. Recorded data in the data registry were also regularly exported and checked by a certified statistician for outliers to verify if data entry error had occurred and were rechecked if needed.

###  Statistical Methods

 The sample size of 368 patients in this study was calculated with an approach without the background of the occurrence of a specific adverse reaction (for example, lipoatrophy). With a power of 80% and 10% drop-out rate, this sample size was estimated to be capable of detecting at least one AR with an incidence of 0.5%.

 It should be noted that the data of all patients were used for analysis for the period of their participation in the study.

 Descriptive analysis was performed. For continuous variables, mean and standard deviation were calculated. In addition, categorical variables were reported by frequency and percentage.

 Safety assessment was the main objective of this study, and it was assessed using the incidence of AEs. A Poisson model using generalized equation estimation and a negative binomial model was performed to assess annual relapse rates (ARRs). Also, a paired t-test was performed to compare the mean of EDSS scores, MusiQoL scores, BDI-II scores, and the mean number of T2 and gad-enhancing lesions.

 For AEs, data were summarized using incidence classified according to System Organ Class (SOC) and Preferred Term (PT) for AEs and SAEs. In other words, to calculate the incidence, patients with any number of AEs were counted only once. The intensity of AEs was graded according to the CTCAE v5.0, and AEs were coded according to the MedDRA SOC and PT (MedDRA Desktop Browser 4.0 Beta). The incidence of grade 3 and 4 AEs was reported. Seriousness was also recorded for all AEs.

 Moreover, causality was evaluated based on the WHO Causality Assessment Scale, and its results were reported by frequency. Also, to compare ARR 12 months before Cinnomer^®^ initiation and 14 months after Cinnomer^®^ treatment, 95% CIs of RR and ARR were estimated based on robust standard errors derived from a Poisson regression model using the generalized estimating equation method and least-square means with the logarithm link function. The reason for using the generalized estimating equation model approach was to analyze the longitudinal count response (here, ARR) to capture the correlation between observations. The negative binomial regression and least-square means with the logarithm link function were used to estimate the RR and ARR for naïve and switcher patients. Also, the proportion of relapse-free patients was determined. The mean values of EDSS Scores, MusiQoL score, and BDI-II at baseline and the end of the study were compared using a paired t-test. Also, changes in the total number of T2 and gad-enhancing lesions at baseline and the end of the study were analyzed by paired *t* test.

## Results

 A total of 368 patients were recruited. Among all subjects, 202 patients completed the study. From those who did not complete the study, 61 patients discontinued Cinnomer^®^.

###  Descriptive Data 

 All 368 enrolled patients were analyzed. Table 1 demonstrates the basic characteristics of the participants. Table S1 presents a summary of the data collected from patients who completed the study, providing a more detailed perspective (see Supplementary file 1).

**Table 1:** Basic Characteristics of the Participants

**Variable**	Value*
Gender	
Female	306 (83.15)
Male	62 (16.85)
Mean age (y)	34.09 ± 8.28
Mean weight (kg)	65.45 ± 11.44
Mean height (cm)	164.20 ± 8.16
Smoking	19 (5.16)
Daily alcohol consumption	2 (0.54)
Pregnant	1 (0.33)
Breast feeding	2 (0.65)
MS treatment history	
Naïve	191 (51.90)
Switcher	177 (48.10)
Disease duration (years)	2.75 (0-8.24)
Number of relapses in the preceding year	
0	183 (49.73)
1	122 (33.15)
2	55 (14.95)
3	7 (1.90)
4	1 (0.27)
Four or more T2 lesions at baseline	149 (79.68%)

* Number (%) for qualitative variables and mean ± standard deviation (SD) for quantitative variables are reported. For disease duration, median (minimum-maximum) is reported.

Table 2 demonstrates the history of those who switched from other drugs to Cinnomer^®^.

**Table 2:** Previous Disease-Modifying Therapy and Reason for Switching to Glatiramer Acetate

**Previous DMT**	**No. (%)**
IFNβ-1a IM	87 (49.15)
IFNβ-1a SC	38 (21.47)
IFNβ-1b	22 (12.43)
GA	14 (7.91)
FTY	11 (6.21)
DMF	5 (2.82)
**Switching reason**	
Adverse events	44 (38.60)
Cost	9 (7.89)
Drug availability	9 (7.89)
Lack of efficacy	33 (28.95)
Physician decision	2 (1.75)
Pregnancy	16 (14.04)
Unknown	63 (55.27)

GA, glatiramer acetate; DMT, disease-modifying therapy; DMF, dimethyl fumarate; FTY, fingolimod.

Table 3 summarizes the reasons for discontinuing Cinnomer^®^. The data on the DMTs that were used after switch from Cinnomer^®^ was available for only 11 patients (6 were changed to rituximab, 2 to fingolimod, 2 to IFNβ-1a IM, and 1 to IFNβ-1a SC). All these patients were included in the final analysis.

**Table 3:** Frequency of Reasons for Cinnomer^®^ Discontinuation

**Discontinuation Reasons**	**No. (%)**
Adverse events	13 (21.31)
Compliance	1 (1.64)
Cost	1 (1.64)
Lack of efficacy	23 (37.71)
Pregnancy	11 (18.03)
Unknown	12 (19.67)

###  Outcome Measures

Table 4 summarizes the study outcomes.

**Table 4:** Clinical and Imaging Outcomes Compared to Before Treatment

	Before *	After **	**Comparison (95% CI)**	***P *** **Value**
ARR	0.78	0.51	RR: 0.65 (0.43,0.98)	0.04
Naïve	0.77	0.27	RR: 0.35 (0.17,0.72)	0.004
Switcher	0.8	0.77	RR: 0.96 (0.59,1.58)	0.89
Switchers (Fingolimod excluded)	0.79	0.51	RR: 0.65 (0.41,1.03)	0.06
EDSS	1.73 ± 1.22 ***	1.52 ± 1.25	Difference: -0.21 (-0.34, -0.08)	0.001
BDI-II score	14.50 ± 11.34	12.11 ± 10.27	Difference: -2.39 (-3.74, -1.03)	< 0.001
MusiQoL Global index score	63.47 **±**15.83	64.07 **±**15.89	Difference: -0.6 (-1.34, 2.54)	0.54
Rejection	74.24 ± 28.96	78.31 ± 25.64		0.04
Psychological Well-being	50.55 ± 25.39	54.40 ± 26.45		0.02
Number of T2 lesions	9.07 ± 6.52	8.75 ± 6.39	Difference: -0.32 (-0.94, 0.29)	0.3
Number of gad-enhancing lesions	0.60 ± 1.24	0.21 ± 0.63	Difference: -0.38 (-0.64, -0.12)	0.004

ARR, annual relapse rate; EDSS, Expanded Disability Status Scale; BDI, Beck Depression Inventory *12 months before Cinnomer^®^ initiation. **14 months after Cinnomer^®^ treatment initiation. ***Median time interval between baseline EDSS and last relapse: 4.68 months.

 By the end of the study, ARR values were 0.27 (95% CI: 0.17,0.43) and 0.77 (95% CI: 0.49,1.22) for treatment naïve and switcher patients, respectively. The ARR of treatment naïve patients was 66% lower compared to DMT switcher patients (RR: 0.34, 95% CI: 0.19,0.62, *P* < 0.001).

 After 6, 10 and 14 months of Cinnomer^®^ initiation, the proportion of relapse-free patients was 85.25%, 77.48%, and 73.76%, respectively.

 The MusiQoL questionnaire (global index score) assessed patients’ QoL, and the results did not show a significant difference comparing before and after Cinnomer^®^ treatment (Table 4). Of all nine dimensions of the MusiQoL questionnaire (activities of daily living, psychological well-being, symptoms, relationships with friends, relationships with family, sentimental and sexual life, coping, rejection, relationship with healthcare system),^5^ the change in psychological well-being and rejection were significant (Table 4). Overall, it can be seen from Table 3 that most of the dimensions showed an increase after the use of Cinnomer^®^.

**Figure 1:**
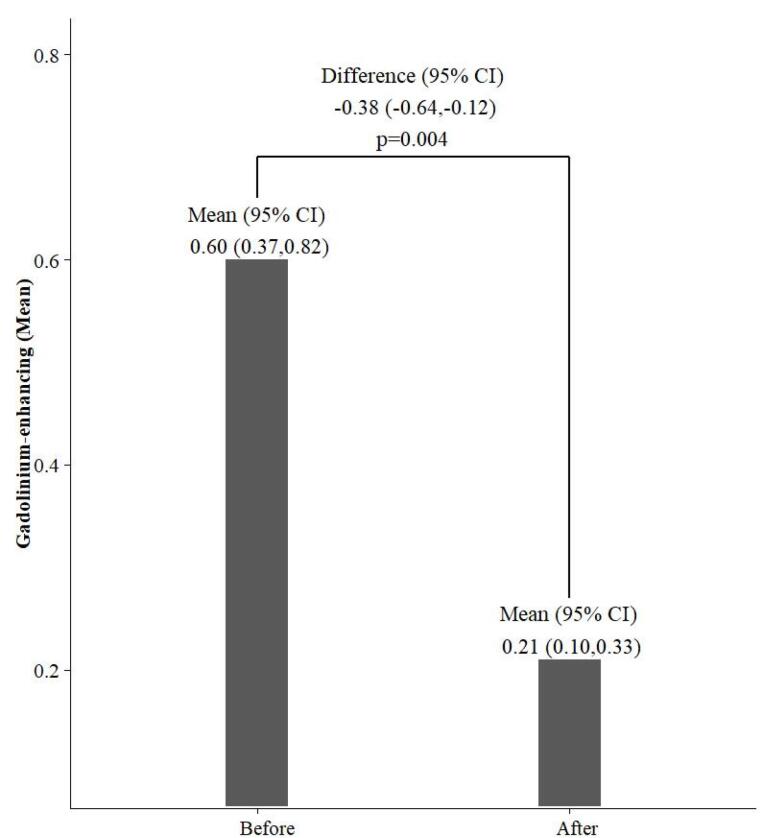
Comparison of the Mean Number of Gad-Enhancing Lesions Before and After Cinnomer^®^

 As shown in Table 4, the change in the mean number of T2 lesions was not significant. However, a significant reduction in the mean number of gad-enhancing lesions after Cinnomer^®^ treatment can be seen (*P* = 0.004) (Figure 1).

## Safety

 A total number of 1008 AEs were reported. The incidence of at least one grade 3 or 4 AE was 35 (9.51%). No SAEs were reported. The incidence of AEs with the number of patients experiencing them are shown in Table 5.

**Table 5:** Incidence of Adverse Events Classified by System Organ Class

**SOC** **System Organ Class**	**PT**	**No. (%)**
Patients with at least one grade 3 and 4 AEs		35 (9.51)
Blood and lymphatic system disorders	At least one event	43 (11.68)
	Anemia	27 (7.34)
	Leukopenia	18 (4.89)
Gastrointestinal disorders	At least one event	52 (14.13)
	Dysphagia	49 (13.32)
	Nausea	3 (0.82)
General disorders and administration site conditions	At least one event	193 (52.45)
	Asthenia	18 (4.89)
	Cold sweat	1 (0.27)
	Influenza-like illness	1 (0.27)
	Injection site reaction	176 (47.83)
	Flushing	49 (13.32)
	Pain	54 (14.67)
Investigations	At least one event	59 (16.03)
	Alanine aminotransferase increased	16 (4.35)
	Aspartate aminotransferase increased	13 (3.53)
	Blood alkaline phosphatase increased	3 (0.81)
	Blood bilirubin increased	6 (1.63)
	Blood creatinine increased	8 (2.17)
	Lymphocyte count decreased	6 (1.63)
	Neutrophil count decreased	22 (5.98)
	Platelet count decreased	3 (0.82)
Metabolism and nutrition disorders	At least one event	13 (3.53)
	Hypernatremia	2 (0.54)
	Hypoalbuminemia	11 (2.99)
Nervous system disorders	At least one event	4 (1.09)
	Dizziness	3 (0.82)
	Headache	1 (0.27)
Psychiatric disorders	At least one event	51(13.86)
	Anxiety	51 (13.86)
Respiratory, thoracic, and mediastinal disorders	At least one event	27 (7.34)
	Dyspnoea	27 (7.34)
Skin and subcutaneous tissue disorders	At least one event	17 (4.62)
	Alopecia	1 (0.27)
	Rash	16 (4.35)
Vascular disorders	At least one event	8 (2.17)
	Vasodilatation	8 (2.17)

AE, adverse event; SOC, System Organ Class; PT, Preferred Term.

 The incidence of grade 3 and 4 AEs is presented in Table 6. Intensity was assessed based on the CTCAE V5.0 criteria. Injection site reaction was the most common grade 3 or 4 AE.

**Table 6:** Incidence of Grade 3 or 4 Preferred Terms

**Preferred Term **	**No. (%)**
Asthenia	1 (0.27)
Injection site reaction	11 (2.99)
Leukopenia	1 (0.27)
Neutrophil count decreased	1 (0.27)
Vasodilatation	8 (2.17)
Anxiety	4 (1.09)
Dysphagia	6 (1.63)
Dyspnea	3 (0.82)
Flushing	6 (1.63)
Pain	4 (1.09)
Rash	1 (0.27)

## Discussion

 This open-label study explored the real-world experience of Cinnomer^®^ treatment in RRMS in Iran.

###  Safety

 The documented AEs were not unexpected for GA and were principally not serious. As anticipated, local injection-site reactions such as erythema and pain were the most frequently reported AEs during the study. The incidence of GA injection site reactions in this study was similar to previous reports.^7-10^ Lebrun-Frenay et al reported local injection site reactions as 73.6% of non-serious AEs.^8^ Elimination of all local injection site-related AEs seems unfeasible, but proper injection techniques may prevent some. Aseptic conditions and helping patient to relax during injection to avoid sudden movement can be helpful. Also, the pain can be reduced by the being at room temperature at the time of injection and administering the drug at the right injection speed.^11,12^ Besides, no SAEs were documented in this study.

###  Effectiveness

 ARR, disease progression, number of MRI lesions, patients’ QoL, and depression were assessed over 14 months.

 ARR is an indicator of the inflammatory aspect of the disease.^13^ Being treated with Cinnomer^®^, patients experienced a significant reduction in ARR. By the end of the study, ARR in treatment naïve patients was 66% lower than switcher patients. The greatest reductions in ARR associated with Cinnomer^®^ were generally seen in treatment naïve patients. Furthermore, the majority of patients were relapse-free with the Cinnomer^®^ treatment during the study period. These results agree with the observed treatment effect of GA on relapse in clinical trials.^14-18^

 In terms of disability progression, the final mean EDSS scores showed significant improvement from baseline to the end of the study. Disability worsening, indicated by increased EDSS, shows the degenerative component of MS. However, the observations in this 14-month study need to be confirmed over time.

 The number of gad-enhancing lesions also significantly decreased in our study during Cinnomer^®^ treatment. These results agree with previous findings.^16,19^

 The prevalence of depression is significantly higher in MS patients compared to those without the disease.^20^ Some studies show that disability accumulation is associated with worse BDI-II scores.^21,22^ Our results show the positive effect of Cinnomer^®^ on total BDI-II score. Tsai proposed that stimulating the production of brain-derived neurotrophic factor in the nervous system may explain the probable effect of GA on depression.^23^

 Among many other MS-specific instruments for assessment of quality of life, the MusiQoL questionnaire has several advantages.^24^ Full involvement of a notable number of MS patients from 15 countries with different MS types and severities, more concise structure compared to other similar questionnaires, and better practicality are some of its positive points.^25^ Patients also improved on measures of QoL. There was a slight increase in mean MusiQoL global index scores from baseline to month 14. Still, QoL improvements were more pronounced and significant in the ‘psychological well-being’ and ‘rejection’ dimensions.

## Limitations

 The open-label setting might be a confounding factor and a possible limitation to the study. However, this is an inherent characteristic of a phase-IV clinical trial in which blinding is not necessarily applied. Similarly, the absence of a control group would be another limitation. A paper-based data collection method (PMS booklets) with a more challenging data monitoring process possibly could cause minor deviations in the results. Since this multicenter-study has been executed in different cities, imaging data were collected in various MRI centers with different protocols and MRI scan readers that might have affected the results. The limited follow-up period (14 months) is another shortcoming that restricts a final conclusion based on the results of this study, so further studies with longer follow-up durations are needed.

## Conclusion

 In conclusion, safety measures were in line with the known profiles of GA. At 14 months after vs. 12 months before Cinnomer^®^ treatment initiation, ARR was significantly lower, and most patients were relapse-free. The number of gad-enhancing lesions significantly reduced during the study. EDSS decreased significantly in comparison to baseline. Furthermore, the global index score and some dimensions of the MusiQoL questionnaire and BDI-II scores showed an improvement, suggesting that Cinnomer^®^ is effective with respect to clinical outcomes and from the patient’s perspective and in reducing MRI-measured MS activity. This study revealed the safety and effectiveness of Cinnomer in the treatment of patients with MS.

## Supplementary Files


Supplementary file 1 contains Table S1.
