# Supplementary material for: Safety and Effectiveness of Cinnomer® on Disease Characteristics, Depression, and Quality of Life of Patients with Multiple Sclerosis: A Phase IV, Post-marketing, Prospective, Multicenter Study
Source: Arch Iran Med. 2023 Nov 1;26(11):647–53. doi: 10.34172/aim.2023.95 (PMC10864943; doi:10.34172/aim.2023.95)
Supplement: Supplementary file 1 — contains Table S1. [file aim-26-647-s001.pdf]

Arch Iran Med. November 2023;26(11):647-S1.
doi 10.34172/aim.2023.95

Table S1. Data of the patients who completed the study

Variable	N = 202*
Gender<br>Female<br>Male	 168 (83.17)<br>34 (16.83)
Mean age (year)	34.60 ± 8.82
Mean weight (kg)	65.77 ± 11.87
Mean height (cm)	164.17 ± 8.33
Smoking	8 (3.96)
Daily alcohol consumption	0 (0.00)
Pregnant	0 (0.00)
Breast feeding	1 (0.60)
MS treatment history<br>Naïve<br>Switcher	 107 (52.97)<br>95 (47.03)
Disease duration (years)	3.06 (0.03 – 8.24)
Number of relapses in the preceding year<br>0<br>1<br>2<br>3	 91 (45.05)<br>75 (37.13)<br>32 (15.84)<br>4 (1.98)
Four or more T2 lesions at the baseline**	135 (83.85%)

*Number (%) for qualitative variables and mean ± standard deviation (SD) for quantitative variables are reported. For disease duration, median (minimum - maximum) is reported.
**Data include patients whose MRI results were available at the baseline only.
